# Effect of fluorescence biomodulation as an adjunct to standard wound management in open wounds in dogs and cats: a prospective, randomised, within-wound controlled clinical trial

**DOI:** 10.3389/fvets.2026.1870940

**Published:** 2026-07-22

**Authors:** María Jiménez Lázaro, Joaquín Sopena Juncosa, Emma Julie Camille Martins, Elena Colombino, Arturo Oliver Guimerá, Verónica Peraile Paños, Laura Mangas Amezqueta, Mónica Rubio Zaragoza

**Affiliations:** 1 Department of Animal Medicine and Surgery, School of Veterinary Medicine, Universidad CEU Cardenal Herrera, Valencia, Spain; 2 Hospital Clínico Veterinario de la Universidad Cardenal Herrera-CEU, CEU Universities, Valencia, Spain; 3 BIOMED SURGERY Research Group (Bioregenerative Medicine and Applied Surgery), School Veterinary Medicine, Universidad Cardenal Herrera-CEU, CEU Universities, Valencia, Spain; 4 Department of Animal Production and Health, Public Veterinary Health and Food Science and Technology, Universidad CEU Cardenal Herrera, Valencia, Spain; 5 Pathology Group, PASAPTA, Facultad de Veterinaria, Universidad Cardenal Herrera-CEU, CEU Universities, Valencia, Spain

**Keywords:** cats, dogs, fluorescence biomodulation, open wounds, photobiomodulation, wound healing

## Abstract

**Background:**

Open wounds are a common clinical problem in small animal practice and are associated with delayed healing, infection, and increased treatment burden. Fluorescence biomodulation (FB), a form of photobiomodulation therapy (PBMT), has shown benefits in several dermatological conditions; however, its efficacy in veterinary open wounds remains unclear.

**Objectives:**

To evaluate the effect of FB as an adjunct to standardised wound management on healing progression in open wounds in dogs and cats using a prospective, randomised, within-wound controlled clinical trial.

**Methods:**

Fifteen client-owned dogs and cats with open wounds of various aetiologies were enrolled. Each wound was divided into two portions: one treated weekly with FB and one untreated control, both receiving standardised wound care based on the TIME framework. Quantitative wound area reduction was assessed by serial planimetric measurements. Qualitative healing parameters were scored weekly by two blinded observers, and semiquantitative histological and immunohistochemical evaluation was performed in surgically treated cases. Paired statistical analyses were used. A time-integrated analysis based on the area under the curve (AUC) of normalised wound area was performed to assess healing kinetics.

**Results:**

No statistically significant differences were observed between treated and control portions for percentage wound area reduction, qualitative healing scores, or semiquantitative histological and immunohistochemical outcomes. Quantitative planimetry showed a heterogeneous response, with seven wounds favouring the treated portion, six favouring the control portion, and two showing equivalent outcomes. Exploratory *post hoc* time-integrated analyses suggested a trend toward earlier wound area reduction in some cases, particularly when treatment was initiated early; however, these findings were not statistically significant. Complete wound closure during follow-up did not differ significantly between portions. Histological analysis showed only small numerical differences between portions, with no statistically significant differences in collagen deposition, angiogenesis, inflammatory response, fibroblast proliferation, or CD31 and Ki67 expression.

**Conclusion:**

Under the conditions tested, FB did not demonstrate a consistent measurable advantage over standardised wound management in open wounds in dogs and cats. Any potential clinical benefit appeared modest and possibly limited to selected wound presentations. Further prospectively stratified studies focused on healing speed and readiness for closure are warranted.

## 1 Introduction

Wound healing is a complex biological process and a frequent clinical challenge in veterinary practice, given the high incidence of cutaneous lesions in dogs and cats. Bite injuries, road traffic accidents, and post-surgical wound complications are among the most common causes of open wounds in companion animals (1–4). Effective wound management is essential to minimize complications such as delayed healing, chronic non-healing wounds, infection, and pain, while also reducing treatment costs and preserving animal welfare (5–8). Over the years, various products and novel therapies have been developed to improve wound healing (9–13).

**Figure 1:**
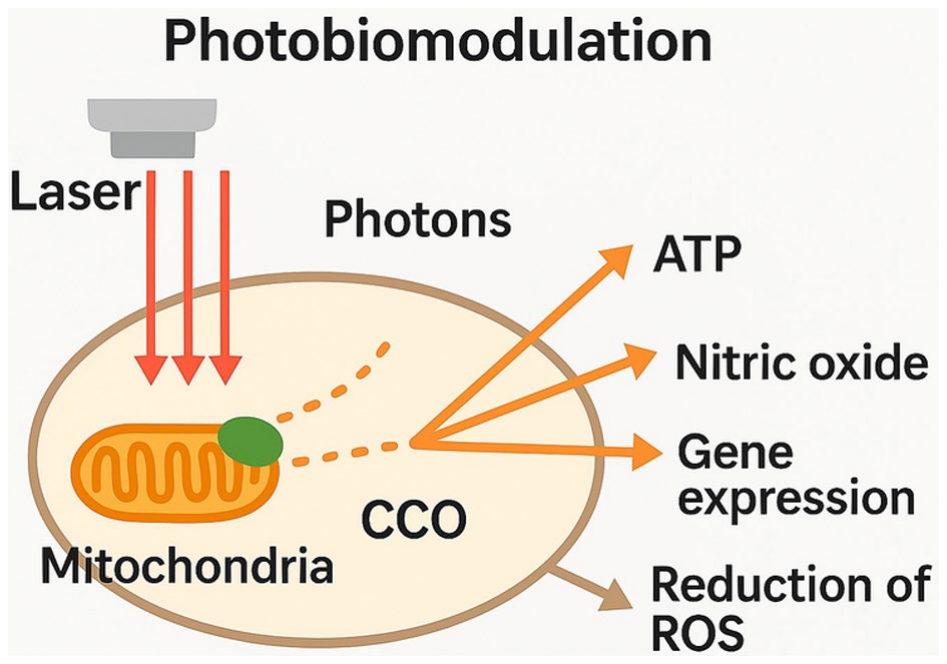
Proposed cellular effects of photobiomodulation therapy (PBMT) on mitochondria. PBMT is thought to stimulate cytochrome c oxidase (CCO), enhancing adenosine triphosphate (ATP) production, modulating reactive oxygen species (ROS), and promoting downstream cellular signalling pathways involved in tissue repair. This figure was created using AI-assisted illustration software.

With the increased knowledge of the healing process, recent treatment options include the use of photobiomodulation therapies (PBMT), a range of non-invasive, non-thermal therapies that use nonionizing light to achieve a therapeutic effect while targeting specific tissues with minimal side effects (14, 15). PBMT uses red and near infrared electromagnetic radiation acting on endogenous light absorbing particles (chromophores) to stimulate the healing of pathologic tissue and to provide pain relief (16–18). At the cellular level, PBMT primarily affects mitochondrial function through cytochrome c oxidase, resulting in increased adenosine triphosphate (ATP) production, nitric oxide release with subsequent improvements in microcirculation, and modulation of reactive oxygen species (ROS), ultimately influencing intracellular signaling pathways involved in cell migration, proliferation, and tissue regeneration (14, 15, 19, 20) (Figure 1).

Among PBMT modalities, fluorescence biomodulation (FB) systems—such as Phovia (Vetoquinol, Paris, France)—utilize fluorescent light energy generated by blue light-emitting diodes (LEDs) in combination with a topical photoconverter substrate (amorphous gel) to stimulate healing. The Phovia system delivers a standardised fluorescent signal without the need for device calibration or parameter adjustment, offering a user-friendly approach for clinical practice (21).

Extensive evidence of the positive effects of PBMT in dermatologic conditions exists both in human and in veterinary patients, including the management of conditions such as otitis externa, canine interdigital pyoderma, deep pyoderma, and perianal fistula (22–27). Among its applications in skin diseases wound healing has also been investigated, and efficacy has been reported in the treatment of chronic, post-surgical wounds and diabetic ulcers (28–30). In dogs, two studies have found better collagen deposition, less inflammation and improved healing of incisional wounds with FB, however one other study in surgically created and open wounds showed no significant effect on healing after laser therapy (31–33).

Although PBMT is frequently used in animals, treatment protocols are not standardised, and controversy exists regarding its clinical efficacy for the treatment of various conditions (34). Initial *in vitro* studies reported significant stimulation of healing of cutaneous wounds and burns in mice with PBMT, however subsequent studies obtained negative results when used in deeper tissues with the same parameters (35, 36). Given that the efficacy of PBMT is dose-dependent, it is now acknowledged that negative outcomes reported in some studies may have resulted from the use of inadequate devices and/or suboptimal therapeutic parameters, emphasizing the necessity of standardised protocols based on the effective photonic dose delivered to the target tissue (14, 37).

Considering current evidence, the potential benefits of FB therapies in the management of clinically challenging open wounds constitute an interesting topic for further research. The objective of the present randomised controlled trial is to evaluate the healing progress in open wounds treated with FB in comparison to a control portion; and to determine if its use significantly reduces time for complete wound healing and epithelialization or time until secondary closure is permitted within the four-week follow-up period.

We hypothesized that fluorescence biomodulation would improve healing measures in open wounds when used as an adjunct to standardised wound management.

## 2 Materials and methods

### 2.1 Study design and ethical approval

The study was approved by the University’s Animal Experimentation Ethics Committee (CEEA). The owners were informed of the nature of the study, and they signed an informed consent form with the possibility to withdraw their animal from the study at any given time. Owners of dogs and cats referred to the Veterinary Teaching Hospital of the CEU Cardenal Herrera University from December 2023 until July 2025 affected with an open wound were given the option to enrol in the study.

The study was conducted for a maximum of 4 weeks in each patient, or less if wound closure occurred earlier or if secondary closure was considered appropriate by the attending surgeon (MJ/EM).

### 2.2 Animals and eligibility criteria

To be included in the study, animals had to present with an open wound due to traumatic tissue loss, burn injuries, surgical wound dehiscence and/or an infection with a total surface from 0.05% up to 6% of the animal’s body surface area (BSA), calculated from the patient’s bodyweight (BW) using the canine and feline conversion tables in BSAVA Small Animal Formulary (38).

All patients included in the research had a full clinical examination, routine blood test (complete blood count, serum biochemistry, and electrophoresis), urine analysis, infectious diseases screening test (*Ehrlichia* and *Leishmania* in dogs; *FIV* and *FeLV* in cats) and wound culture.

Animals were excluded from the study if the wound was closed surgically within the initial 72 h of presentation; if they had received previous treatment with laser therapy or FB; if the wound was associated with an oncological surgery with incomplete surgical margins; and if the wound was associated to an orthopaedic condition with the presence of metal implants.

### 2.3 Treatment protocol and wound management

For each animal included in the study, the full length of the wound was divided into two halves at the time of presentation, A (proximal or left), and B (distal or right). Because irregular wound geometry often precluded exact symmetrical partitioning, subsequent quantitative analyses were based on normalised change over time and paired within-case comparisons. Although the split-wound design allowed each patient to act as its own control, it cannot be excluded that biological effects induced by fluorescence biomodulation extended beyond the designated treatment area, particularly in smaller wounds. The treatment (T) and control (C) portions were randomly assigned by a blind member of the staff outside of the study. The original size of the longitudinal wound axis was noted and marked in the animal by using a skin staple to maintain a reference throughout the entire duration of the study.

Once a week the T portion of the wound was treated with FB therapy, while the C portion of the wound only received a standardised wound therapy protocol. Patients were positioned to keep the wound accessible, with the head turned away from the light source. Sedation could be used when necessary to ensure safe handling and patient comfort. Immediately before use, the exogenous chromophore was mixed into the carrier gel and stirred until a homogeneous orange coloration was obtained; the container was kept covered until application. A uniform gel layer (approximately 2 mm thick) was applied over the entire wound surface using a spatula and illuminating it with a blue LED device that delivers noncoherent blue light with a peak wavelength between 440 and 460 nm and a power density of between 55 and 129 mW/cm2, for 2 min, at approximately a 5 cm distance. After illumination, the gel was gently removed using sterile gauzes immersed in sterile saline solution and a second illumination performed, 1 min apart. All personnel present wore protective eyewear during illumination.

All animals were treated with a standardised wound therapy protocol based on the TIME strategy to prepare the wound bed (39) (Table 1). Once an adequate wound bed was obtained, the attending surgeon decided on surgical closure, or second intention healing considering wound, patient, and client related factors.

**Table 1:** Standardised wound therapy protocol based on the TIME strategy for wound bed preparation and clinical management according to wound healing phase.

Phase of wound healing	Aspect of the wound	Treatment strategy
T (Necrotic or devitalized tissue)	Black necrotic and/or slough tissue	Surgical debridement Pressure cleaning with sterile Ringer’s lactate or saline solution Polyhexanide-betaine irrigation Absorbent non-adherent foam dressing Frequency of wound therapy every 24–48 h.
I (Inflammation and infection)	Contaminated wound with purulent exudate Dark or pale red, inflamed and exuberant granulation tissue	Pressure cleaning with sterile Ringer’s lactate or saline solution Polyhexanide-betaine irrigation Absorbent non-adherent foam dressing Frequency of wound therapy every 24–48 h
M (Moisture and granulation)	Bright red, moist and flat granulation tissue Adhered and active epithelial edges	Irrigation with sterile Ringer’s lactate or saline solution Polyhexanide-betaine irrigation Surgical therapy; or second intention healing with active dressings including hydrogel and an absorbent non-adherent foam dressing Frequency of wound therapy every 3 to 5 days
E (Epithelial, edge, advancement)	Active epithelial edges bridging the wound bed and presence of wound contraction	Irrigation with sterile Ringer’s lactate or saline solution Polyhexanide-betaine irrigation Surgical therapy; or second intention healing with active dressings including hydrogel and an absorbent non-adherent foam dressing Frequency of wound therapy every 5 to 7 days

### 2.4 Microbiological assessment and medical management

Antimicrobial therapy was initiated when indicated with cefazolin at 22 mg/kg every 12 h IV (Cefazolin, Normon, Madrid, Spain) or cefalexin 15 mg/kg every 12 h VO (Cefaseptin, Dechra, Shrewsbury, United Kingdom) and adjusted later based on culture and sensitivity results when necessary. All patients received analgesia with meloxicam at the initial dose of 0.2 mg/kg S.C. (Meloxidolor, Dechra, Shrewsbury, United Kingdom), followed by 0.1 mg/kg P.O. q24h in dogs or 0.05 mg/kg P.O. q24h in cats (Metacam, Boehringer Ingelheim, Spain) and/or paracetamol at 10 mg/kg IV or PO every 8 to 12 h in dogs (B. Braun, Melsungen, Germany).

### 2.5 Outcome measures

#### 2.5.1 Clinical and planimetric outcomes

Outcome measures included percentage of reduction of wound size relative to day 0 and between each week of therapy.

**Figure 2:**
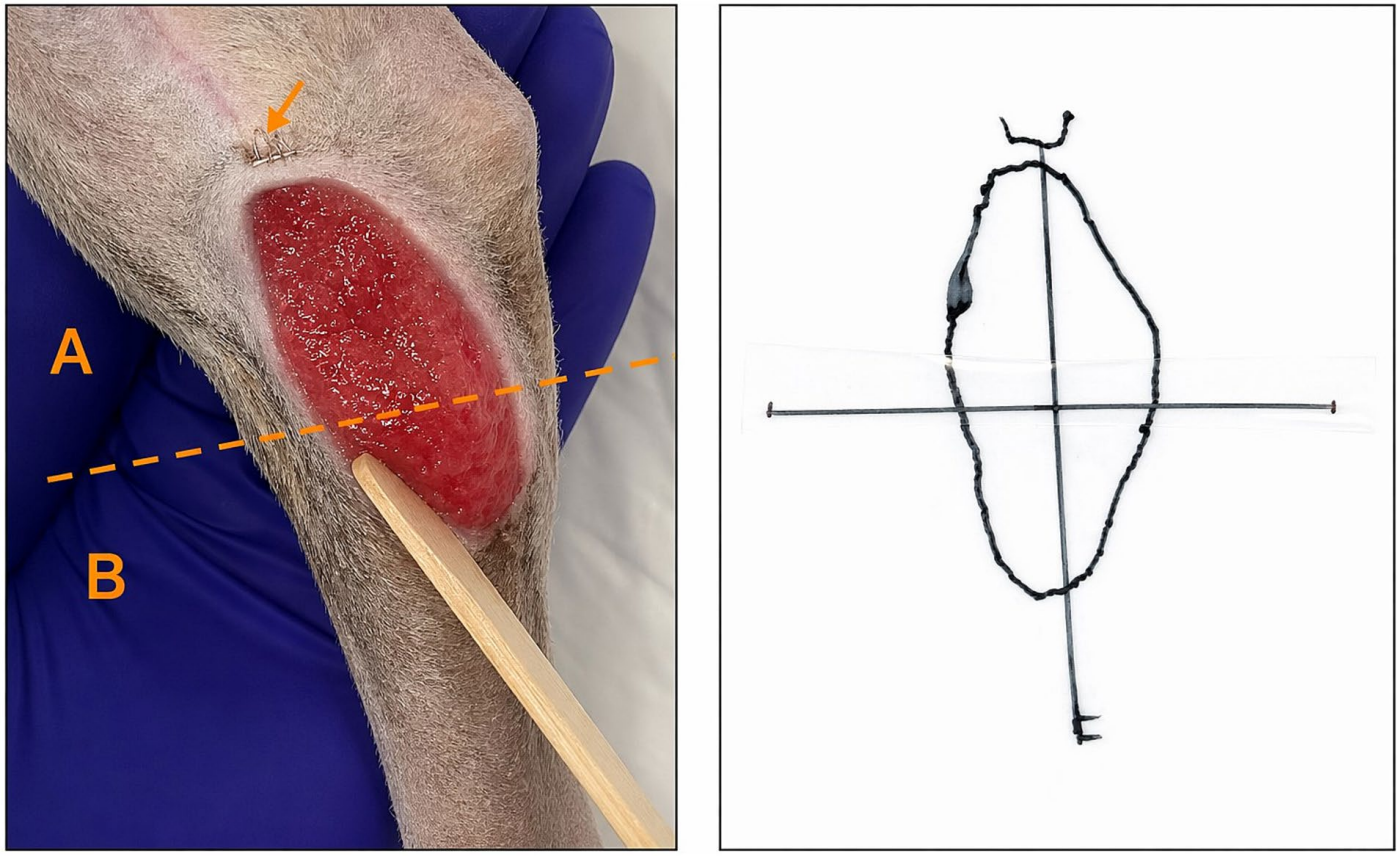
Representative example of split-wound allocation and planimetric area assessment in Case 4 at week 3. The skin staple (arrow) indicates the reference point used to preserve the original longitudinal wound axis throughout follow-up. The wound was divided into two portions: A, corresponding to the control portion, and B, corresponding to the fluorescence biomodulation-treated portion. The accompanying tracing map was used for digital planimetric measurement of wound area. Layout and annotations were refined using AI-assisted editing software without altering the original clinical content.

Quantitative assessment of the wound size was obtained using digital planimetry software (Photoshop CS5 extended version, Adobe Systems Inc., Mountain View, CA, USA) on a map draft taken from the wound by the attending surgeon at time of presentation and in each week for 4 weeks, or until wound closure, or surgical intervention (Figure 2).

#### 2.5.2 Macroscopic scoring

Outcome measures included weekly scoring of necrotic tissue, inflammation and infection, granulation tissue and epithelium observed during therapy.

Semiqualitative data was obtained weekly from wound assessments by two blind observers (VP/LM) using photographs and video records taken every week during wound treatment and before FB therapy. To standardise the wound assessment, a 0–3 scale was designed for the study adapted from the TIME concept created by the European Wound Management Association based on the wound bed preparation framework (39). The observer evaluated the presence of exudate and necrotic tissue, inflammation and infection, granulation tissue, and epithelium giving weekly wound scores (Table 2). The macroscopic scoring system was specifically developed for the purposes of this study based on the TIME framework and was intended as a structured clinical assessment tool rather than a formally validated wound scoring system.

**Table 2:** Semiquantitative scoring system for macroscopic evaluation of wounds.

SCORE	0	1	2	3
T (Necrotic or devitalized tissue)	No devitalized tissue	Black necrotic and/or slough tissue	Yellow or brown necrotic adipose tissue	Skin and subdermal necrosis The muscle tissue appears brown and dull Thrombosed blood vessels (black colour), no pulse, or bleeding from the wound
I (Inflammation and infection)	No exudate	None/minimal exudate	Moderate amount of exudate and/or purulent discharge	Very contaminated exudative wound with purulent discharge and/or subdermal abscesses
M (Moisture, granulation)	No granulation tissue, the wound bed is covered epithelium	Bright red and moist granulation tissue Flat granulation bed, levelled to the epithelial edges	Inflamed and exuberant granulation tissue raising above the epithelial edges	Dark or pale red granulation tissue
E (Epithelial, edge, advancement)	100% of the wound is covered by epithelium	Active epithelial edges bridging the wound bed and presence of wound contraction	Adhered and active epithelial edges advancing towards wound surface	Lack of adhesion or eversion of epithelial edges

Total wound score = sum of all parameters (range 0–3). Lower scores = better healing.

#### 2.5.3 Histopathological and Masson’s trichrome assessment

In seven wounds requiring surgical closure, biopsies were collected from both the control (C) and treated (T) portions during the same anaesthetic event immediately prior to closure. Biopsy sampling was not performed in wounds healing by second intention to avoid unnecessary tissue trauma.

Tissue samples from each portion were obtained using a sterile 4-mm biopsy punch and subsequently fixed in 10% buffered formalin, embedded in paraffin, sectioned at 4 μm, and stained with haematoxylin–eosin (H&E) and Masson’s trichrome. Masson’s trichrome staining was additionally used to semiquantitative assess collagen fibre deposition and extracellular matrix organization between treated and control wound portions.

Histological evaluation was performed by two independent, blinded pathologists using (EC/AO) a semiquantitative scoring system adapted from previously published veterinary wound-healing models (9, 32, 40, 41) (Table 3). The parameters assessed included inflammatory infiltrate, fibroblast proliferation, collagen deposition, and angiogenesis. Each parameter was scored on a 0–3 scale, yielding a maximum composite score of 12.

**Table 3:** Semiquantitative histological scoring system for cutaneous wound healing.

Parameter	0	1	2	3
Inflammation (Evaluated by scoring neutrophils, eosinophils, macrophages, lymphocytes, plasma cells, and mast cells detected in 10 high power fields (HPF) (400x))	>31 cells/field	11–30 cells/field	3–10 cells/field	<3 cells/field 3–10
Fibroblasts	<3 fibroblasts/field 400x	3–10 fibroblasts/field 400x	11–30 fibroblasts/field 400x	>31 fibroblasts/field 400x
Angiogenesis	<3 capillary buds/field 400x	3–10 capillary buds/field 400x	11–30 capillary buds/field 400x	>31 capillary buds/field 400x
Collagen deposition	Absent	Sparse, disorganized fibres	Moderately dense, partially organized	Dense, mature, well-organized bundles

Total score = sum of items (range: 0–12). Higher scores indicate more advanced healing.

#### 2.5.4 Immunohistochemical evaluation

In addition, immunohistochemical analysis was performed on biopsy-derived tissue sections using antibodies against CD31 and Ki67 to assess angiogenesis and cellular proliferation, respectively. All slides were evaluated by the same two independent, blinded pathologists (EC/AO) using a semiquantitative scoring system adapted from previously published studies, as detailed in Table 4 (32, 41).

**Table 4:** Semiquantitative immunohistochemistry scoring system for wound healing.

Parameter	0	1	2	3
Angiogenesis (CD31)	Absent	Few vessels	Moderate vessel density	Extensive neovascularization
Ki67 (Proliferation index)	<5% labeling	5–15% labeling	15–30% labeling	>30% labeling

Total IHC Score: sum of each marker (range 0–6). A higher total indicates more advanced tissue remodeling.

#### 2.5.5 Statistical analysis

*A priori* sample size calculation was performed before study initiation. Statistical analyses were performed using StataNow/MP 19.5 for Windows (64-bit x86-64) (StataCorp LLC, College Station, TX, USA). Based on expected clinically relevant differences in wound area reduction between treated and control portions, and considering a paired within-wound design, a minimum sample size of nine patients was estimated to provide adequate statistical power for the primary outcome. The final number of cases enrolled therefore exceeded the minimum required sample size. The primary endpoint was paired percentage wound area reduction over follow-up. Secondary endpoints were qualitative scores, closure status, histology, immunohistochemistry, and exploratory AUC analyses. Statistical significance was set at *p* < 0.05. Given the exploratory nature of the study and limited sample size, no formal multiplicity adjustment was applied, and secondary analyses were interpreted cautiously. Results were also interpreted with caution due to the small sample size and the presence of non-evaluable data.

Normality of continuous variables (percentage wound area reduction, paired treatment-control differences, baseline wound area, and wound duration at inclusion) was assessed using the Shapiro–Wilk test. As normality assumptions were not consistently met, and several outcomes were ordinal, non-parametric methods were selected for inferential analyses.

Quantitative, qualitative, and histological outcomes were analysed using paired comparisons between the treated and control portions of each wound. Wound areas were normalised to their respective baseline measurements to account for occasional baseline asymmetry between portions. Percentage reduction of wound area was compared using the Wilcoxon signed-rank test, and an exploratory assessment was performed to evaluate the influence of baseline wound size through Spearman rank correlation analysis and stratification by median surface area. For paired outcomes analysed using the Wilcoxon signed-rank test, treatment effects were additionally described using the median paired difference (T−C) with 95% confidence intervals and the rank-biserial correlation as a measure of effect size. The rank-biserial correlation was calculated from the Wilcoxon signed-rank test as the difference between the sums of positive and negative ranks divided by the total sum of ranks.

To further assess differences in the temporal pattern of wound area reduction, an exploratory/ *post hoc* time-integrated analysis was conducted using serial weekly planimetric measurements (Cs1–Cs4 and Ts1–Ts4). Wound areas were normalised to the initial measurement of each portion to account for differences in starting size, and the area under the curve (AUC) of normalised wound area over time was calculated. Paired differences in AUC between treated and control portions were analysed using the Wilcoxon signed-rank test. An additional exploratory stratification was performed according to wound duration at inclusion (≤7 days vs. > 7 days). Complete wound closure during the observation period (area = 0 cm^2^) was analysed as a paired binary outcome using the exact McNemar test.

Qualitative wound-healing parameters (necrosis, inflammation, granulation tissue, and epithelialization), scored on an ordinal 0–3 scale by two blinded observers, were summarized by averaging observer scores for each case and week, and subsequently described as weekly group means. Missing values due to complete healing were treated as non-evaluable. Paired comparisons were conducted using the Wilcoxon test, and interobserver agreement was assessed with Spearman’s rank correlation.

Semiquantitative histological and immunohistochemical scores were compared between portions using the Wilcoxon signed-rank test after averaging the scores of two blinded pathologists. Individual histological parameters (inflammatory infiltrate, fibroblast proliferation, collagen deposition, and angiogenesis), as well as immunohistochemical markers (CD31 and Ki67), were also analysed using the same paired non-parametric approach. Immunohistochemical variables were assessed both individually and as a composite score. Interobserver agreement was evaluated using Spearman’s correlation.

## 3 Results

### 3.1 Study population

Of the 46 animals presenting with open wounds, 15 met the inclusion criteria and were enrolled in the study (Table 5), comprising eight dogs (53%) and seven cats (47%). The cohort included eight males (53%) and seven females (47%). All cats except one mixed-breed individual were Domestic Shorthairs, while the canine population represented various breeds, including mixed breeds, German Shepherd, Border Collie, Pinscher, and Dalmatian.

**Table 5:** General characteristics of the study population.

Case	Sp	Br	Sex	Age_mo	Wt_kg	BSA_cm^2^	Wound_d	AB	NSAID	Inf_Dz	Cult	Cause	Comorb
1	Dog	Mix	M	120	5	3,000	2	Y	N	Neg	Pos	DEHIS	Y
2	Dog	GSD	M	96	28	9,300	14	Y	N	LEISH	Pos	BITE	Y
3	Dog	BC	F	60	18	6,900	41	Y	Y	Neg	Pos	BITE	N
4	Cat	DSH	F	36	2.5	1840	2	N	N	Neg	Pos	UNK	N
5	Cat	DSH	F	50	3.5	2,310	42	Y	N	Neg	Pos	PYOT	Y
6	Dog	PIN	F	144	4	2,500	7	Y	N	Neg	Pos	THERM	N
7	Dog	Mix	F	60	19	7,200	7	Y	N	Neg	Pos	DEHIS	N
8	Dog	Mix	M	168	30	9,800	21	Y	Y	Neg	Pos	ABSC	Y
9	Cat	DSH	M	36	4	2,520	NA	N	Y	Neg	Pos	UNK	N
10	Cat	DSH	M	36	4	2,520	NA	N	Y	Neg	Pos	UNK	N
11	Cat	DSH	M	7	4.5	2,730	4	N	Y	Neg	Pos	UNK	N
12	Dog	Mix	M	98	35	10,800	1	Y	N	Neg	Pos	THERM	N
13	Cat	Mix	M	59	4.5	2,730	3	N	N	Neg	Pos	UNK	N
14	Dog	DAL	F	134	35	10,800	NA	N	N	Neg	Pos	SELF	Y
15	Cat	DSH	F	60	4.5	2,730	NA	N	N	Neg	Pos	PYOT	Y

Sp = Species; Br = Breed; Sex = M (male), F (female); Age_mo = Age in months; Wt_kg = Weight (kg); BSA_cm2 = Body surface area (cm^2^); Wound_d = Wound duration in days; AB = Antibiotics prior to study enrolment (Y/N); NSAID = NSAIDs prior to study enrolment (Y/N); Inf_Dz = Infectious disease results (Neg = negative; LEISH = Leishmania positive); Cult = Bacterial culture result; Cause = UNK (unknown), BITE (bite wound), THERM (thermal abrasion or road traffic trauma), DEHIS (post-surgical complication or traumatic dehiscence), ABSC (abscess), PYOT (pruritic dermatitis / pyotraumatic lesion), SELF (self-inflicted lesion or self-trauma); Comorb = Comorbidities (Y/N).

Patient age ranged from 7 to 168 months (mean: approximately 78 months), and body weight ranged from 2.5 to 35 kg, corresponding to body surface areas of 1,840–10,800 cm^2^ according to BSAVA reference values (38). The duration of the wound prior to presentation varied between 1 and 42 days (mean: 13.7 days, excluding missing values).

Prior to enrolment, 53% of patients (*n* = 8) had received antibiotic therapy and 47% (*n* = 7) had been treated with anti-inflammatory drugs. Infectious disease screening identified one dog (Case 2) positive for *Leishmania* infection, while all other patients tested negative for for *Ehrlichia*, *FIV*, and *FeLV*. Treatment for leishmaniosis was initiated concurrently with wound management and continued throughout the study period.

Bacterial culture was positive in all cases (100%). Culture results identified a diverse microbial population, frequently involving mixed infections (Supplementary Table S1). Antimicrobial susceptibility profiles were variable; however, first-line EMA category D agents were active against all isolates in over half of the cases (8/15) (42). Susceptibility to category C antimicrobials, but not to category D, was observed in six cases. Isolates from one wound demonstrated susceptibility exclusively to category B agents, without shared sensitivity to lower-tier drugs. Notably, seven cases lacking susceptibility to category D had received antibiotic therapy prior to presentation and sample collection at our centre. Two isolates exhibited multidrug-resistant profiles; both had received prior combined antimicrobial therapy involving EMA category B and C agents and demonstrated susceptibility to only a single antimicrobial drug, markedly restricting therapeutic options. Although category A agents demonstrated *in vitro* activity in a small number of isolates, they were not required for clinical management.

The aetiology of the wounds was diverse, including unknown origin (*n* = 5), bite injuries (*n* = 2), thermal or trauma-associated injuries (*n* = 2), traumatic or post-surgical dehiscence (*n* = 2), abscess-associated lesions (*n* = 1), and pruritic or self-inflicted lesions (*n* = 3). Comorbidities were present in six patients (40%).

### 3.2 Quantitative wound area assessment

**Figure 3:**
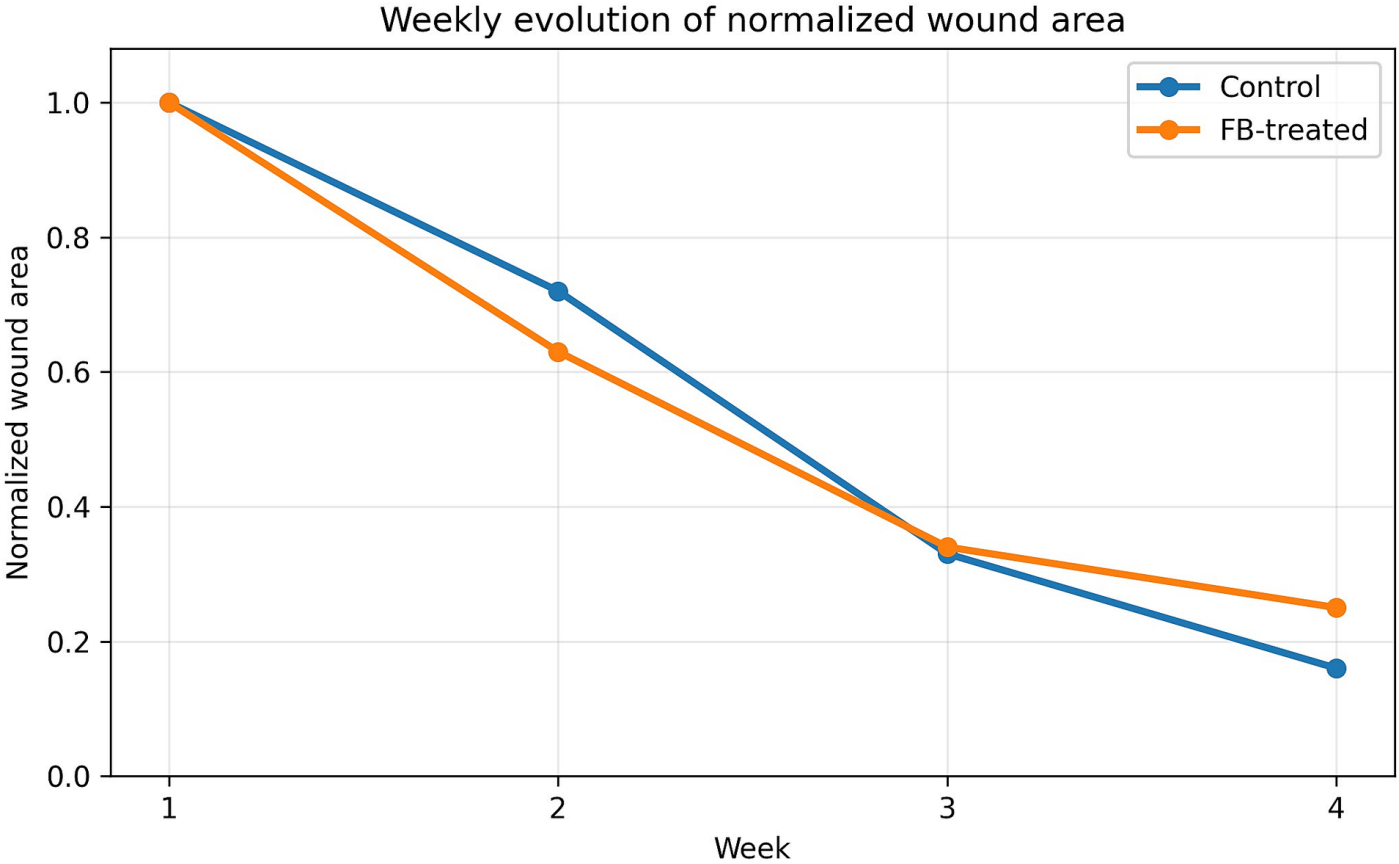
Weekly evolution of normalised wound area in treated and control portions. Mean normalised wound area over the four-week follow-up period for the control portion (blue) and the fluorescence biomodulation–treated portion (orange). Wound areas were normalised to the initial measurement of each portion (week 1 = 1). Both portions showed a progressive reduction in wound area over time, with no clear separation between curves across the study period. The number of evaluable cases decreased over time as wounds reached complete closure. This diagram was created using AI-assisted illustration software.

Quantitative planimetric analysis of wound surface area showed a heterogeneous pattern of response. Among the 15 cases evaluated, seven wounds exhibited a greater percentage reduction in the FB-treated portion, two showed equivalent reduction in both portions, and six displayed greater reduction in the untreated control portion (Figure 3). Individual planimetric measurements for each wound portion and time point are reported in Supplementary Table S2.

Because initial wound size differed markedly between cases, an exploratory analysis was conducted to assess its potential influence on percentage reduction. The first seven enrolled cases had a larger mean combined baseline wound area (34.31 cm^2^) compared with the last eight wounds (6.85 cm^2^), creating a fivefold size difference between subgroups. Reductions were analysed in relation to baseline wound area. Correlations between baseline size and percentage reduction were weak for both the control portion (Spearman’s *ρ* = 0.08; *p* = 0.787) and the treated portion (Spearman’s ρ = −0.37; *p* = 0.176). Furthermore, stratification around the observed median combined baseline wound area (9.89 cm^2^) showed mean reductions of 71.8% in smaller wounds and 69.8% in larger wounds. These findings suggest that baseline wound size alone does not account for the variability observed in treatment response. However, given the limited sample size and heterogeneity in wound presentation, a modest confounding influence cannot be fully excluded. The median paired difference in percentage wound area reduction between treated and control portions (T − C) was 0.0% (95% CI: −23.6 to 27.7%). The difference between portions was not statistically significant (Wilcoxon signed-rank: z = 0.54, *p* = 0.589, two-sided; exact *p* = 0.618).

An exploratory analysis was conducted using the serial planimetric measurements collected weekly (Cs1–Cs4 and Ts1–Ts4) to evaluate whether the treated portion showed earlier or faster area reduction over time. Wound areas were normalised to the first measurement in each portion (Cs1 for the control portion and Ts1 for the treated portion) so that comparisons reflected change over time rather than differences in starting size. A time-integrated metric (normalised area AUC) was calculated across the available weekly timepoints up to the last evaluable planimetric assessment; clinically, a lower AUC indicates that the portion spent less time with a larger open surface, consistent with faster reduction across the follow-up period.

Using this approach, AUC favoured the treated portion in 8/15 cases (cases 2, 4, 6, 7, 9, 10, 11, and 13). The median paired AUC difference (AUC_T−AUC_C) was −0.21 (mean 0.02). The 95% confidence interval for the median paired difference was −0.40 to 0.46, indicating no meaningful difference in healing kinetics between portions. The associated effect size was negligible (rank-biserial correlation = −0.05), and the difference was not statistically significant (Wilcoxon signed-rank: z = −0.17, *p* = 0.865, two-sided; exact *p* = 0.890).

An exploratory stratification by wound duration at inclusion was also performed using the available duration data. Among wounds present for ≤7 days (*n* = 7), AUC favoured the treated portion in 5/7 cases. The Wilcoxon signed-rank test showed no statistically significant difference between portions (z = −0.51, *p* = 0.612; exact *p* = 0.688). Among wounds present for >7 days (*n* = 4), AUC favoured the treated portion in 1/4 cases, and no significant difference was observed (z = 1.10, *p* = 0.273; exact *p* = 0.375).

Finally, a clinically meaningful milestone was examined: complete closure recorded during the observation period (planimetric area = 0 cm^2^ at any assessment). Closure occurred in the treated portion but not the control portion in 3 cases (6, 7, and 10), whereas the reverse occurred in 1 case (15); both portions reached 0 cm^2^ within the observation window in 2 cases (11 and 14). The discordant-pair comparison was not statistically significant (exact McNemar: *p* = 0.625, two-sided).

### 3.3 Qualitative wound assessment

**Figure 4:**
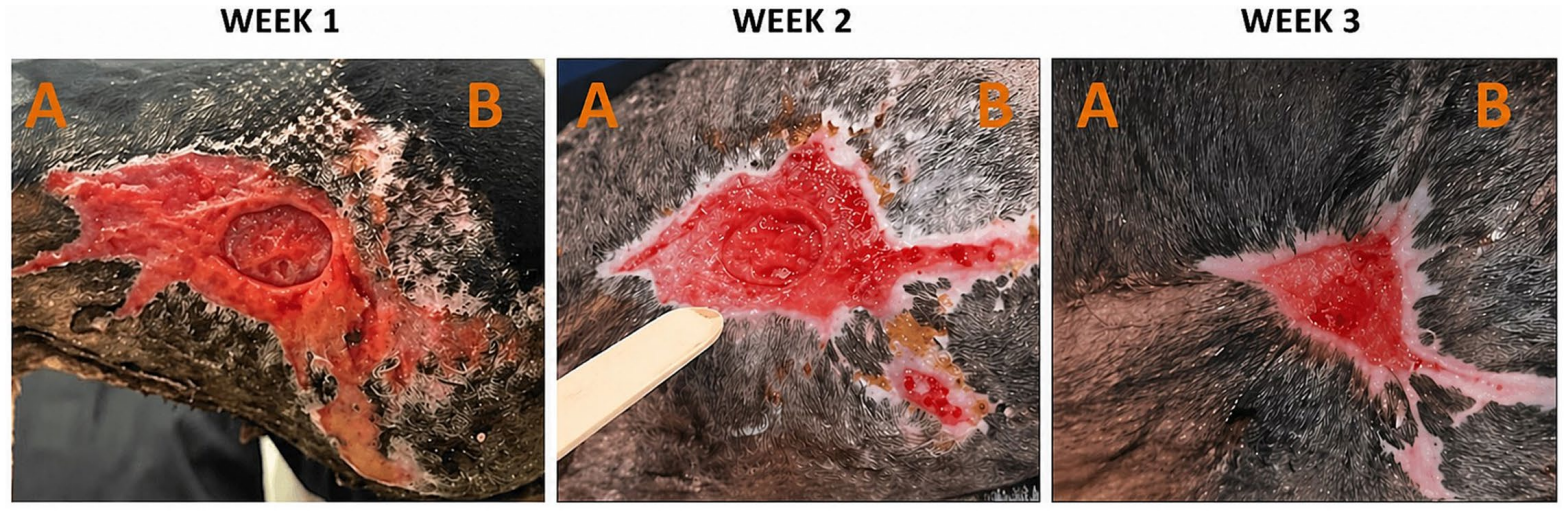
Representative photographic evolution in Case 6 over a 3-week period. Portion A corresponded to the control side, whereas portion B received therapy with fluorescence biomodulation. Layout and annotations were refined using AI-assisted editing software without altering the original clinical content.

Across all four qualitative parameters (necrotic tissue, inflammation, granulation tissue, and epithelialization) and throughout the 4 weeks of evaluation, no consistent or statistically significant differences were detected between the treated and control portions of the wounds (Supplementary Tables S3, S4). Mean weekly scores evolved similarly in both groups, with reductions in necrotic tissue, inflammation, granulation tissue, and epithelialization scores over time, following the expected trajectory of wound healing, as illustrated in a representative clinical case (Figure 4). Wilcoxon paired tests yielded non-significant *p*-values for all comparisons (all *p* ≥ 0.08), indicating no measurable difference between portions using this ordinal scale within the observation period. The magnitude of paired differences was small and showed no consistent pattern favouring either portion across the weekly assessments.

Inter-observer agreement varied across parameters: it was higher for granulation tissue and epithelialization, but lower for necrotic tissue and inflammation, contributing to variability in individual scores. In later weeks, the number of evaluable cases decreased because many wounds had already fully epithelialized, which further reduced the statistical power of the analysis.

### 3.4 Semiquantitative histological and Masson’s trichrome assessment

Semiquantitative histological scores were available for seven surgically treated wounds; however, in one case the treated portion was considered non-evaluable by both observers and was therefore excluded from paired analysis (Supplementary Table S5). Among the remaining six evaluable cases, the mean composite histological score was slightly higher in the control portion than in the treated portion (6.43 vs. 6.17), although the magnitude of this difference was small.

A Wilcoxon signed-rank test showed no statistically significant difference between portions (*p* = 0.914). The median paired difference in total histological score (T−C) was 0.0 points (95% CI: −0.95 to 1.40), indicating no meaningful treatment effect. The associated effect size was negligible (rank-biserial correlation = 0.05). Interobserver agreement for the total histological score was modest, with variable concordance across cases.

**Figure 5:**
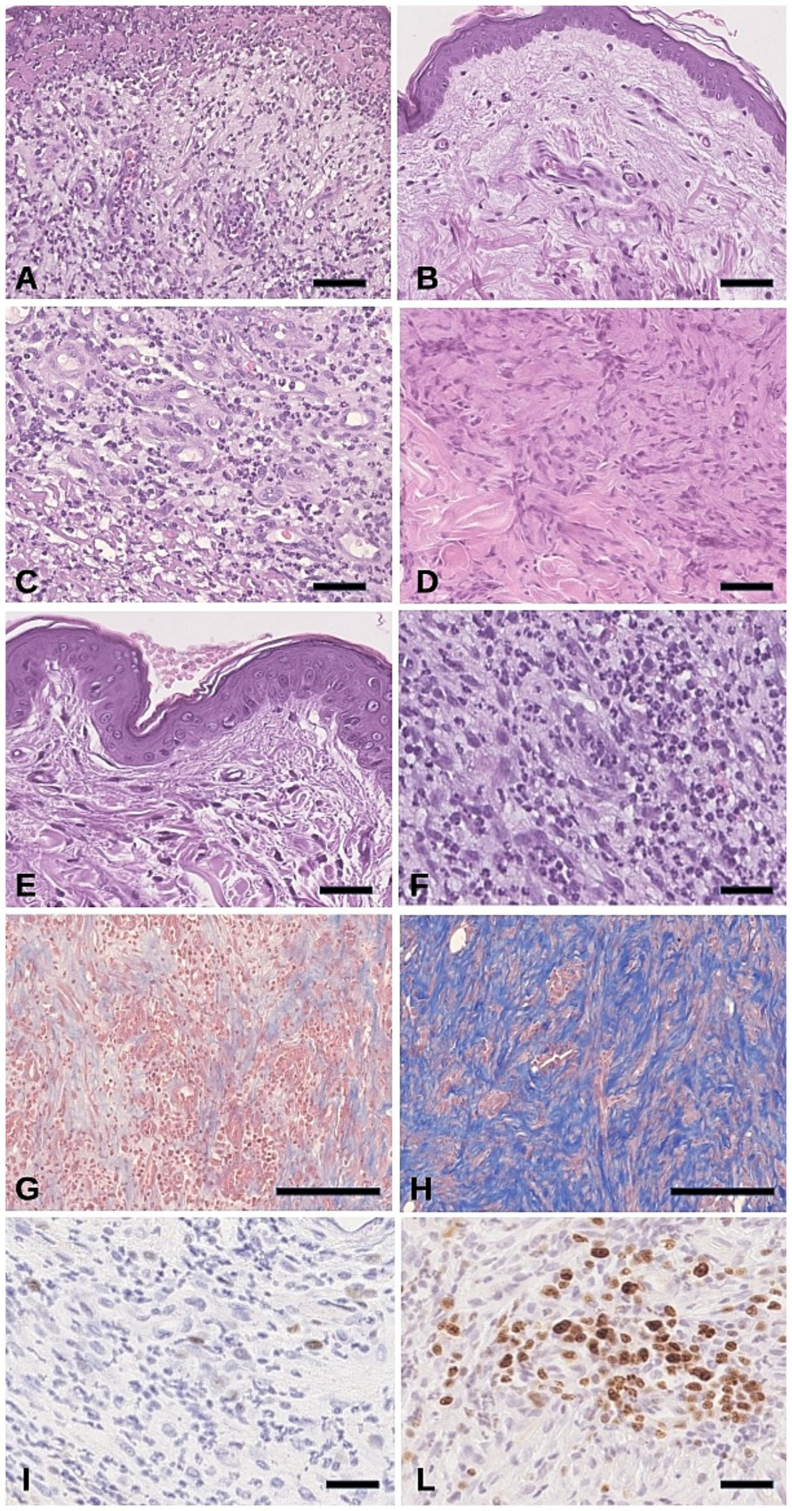
Main histological, histochemical and immunohistochemical findings in the analysed samples. Collagen deposition in control group (**A**, grade 0) and treated group (**B**, grade 2), 20x, Haematoxylin and Eosin (H-e); Fibroblast proliferation in control group (**C**, grade 1) and treated group, (**D**, grade 3), 20x, H-e; Inflammatory infiltrate in control group (**E**, grade 0) and treated group (**F**, grade 3), 20x, H-e; Masson’s trichrome staining in control group (**G**, grade 0) and treated group (**H**, grade 2) 10x (lower magnification; scale bars correspond to a larger distance than in the remaining panels); Ki67 immunohistochemistry in control group (**I**, grade 0) and treated group (**L**, grade 2), 20x.

When analysed individually, fibroblast proliferation showed slightly higher mean scores in treated portions than in control portions (2.50 vs. 2.43) (Figures 5C,D). Inflammation scores were similar between portions (0.92 vs. 0.93) (Figures 5E,F), whereas angiogenesis scores were marginally higher in control portions (1.07 vs. 0.83). Collagen deposition scores were also comparable between portions (2.00 vs. 1.92) (Figures 5A,B). None of these paired comparisons reached statistical significance (all *p* > 0.30).

Masson’s trichrome staining scores were similar between groups, with a small numerical tendency favouring the control portions (1.79 vs. 1.58). The median paired difference (T−C) was 0.0 points (95% CI: −1.40 to 1.40), with no detectable effect (rank-biserial correlation = 0.00), and no statistically significant difference between portions (Wilcoxon signed-rank test, *p* = 1.000), indicating no measurable difference in collagen fibre organization or matrix staining intensity between portions (Figures 5G,H).

### 3.5 Semiquantitative Immunohistochemical assessment

Semiquantitative immunohistochemical scores were available for seven surgically treated wounds. In one case, one observer considered one portion non-evaluable, whereas the second observer provided a score; therefore, the available score was retained for analysis, and all seven cases were included in the paired comparison (Supplementary Table S5). After averaging observer scores where both were available, the mean composite immunohistochemical score was similar between the treated and control portions (0.46 vs. 0.57, respectively), and the magnitude of this difference was small.

A Wilcoxon signed-rank test showed no statistically significant difference between portions (*p* = 0.665). The median paired difference (T−C) was 0.0 points (95% CI: −1.19 to 0.84). The associated effect size was small (rank-biserial correlation = −0.20), favouring the control portion. Interobserver agreement for the overall immunohistochemical score was modest, with variable concordance among cases.

Separate marker analysis showed very low CD31 scores in both portions (0.00 vs. 0.07; *p* = 0.317), indicating no detectable difference in angiogenic immunolabelling. Ki67 scores were also similar between portions (0.46 vs. 0.50; *p* = 0.796), and no statistically significant differences were identified for either marker (Figures 5I,L).

## 4 Discussion

No consistent associations were identified between treatment response and baseline clinical variables—including wound aetiology, comorbidities, and prior antibiotic or anti-inflammatory therapy—although these *post hoc* exploratory analyses were limited by the small sample size. A numerical tendency toward earlier wound area reduction was more frequently observed in wounds treated with FB at an early stage.

Antimicrobial stewardship represents a critical component in the management of open wounds, particularly in the context of increasing bacterial resistance and the EMA classification framework (42). In the present study, culture and susceptibility testing revealed heterogeneous microbial populations and variable resistance patterns; however, clinical resolution was achieved in all cases through appropriate wound management without reliance solely on antimicrobial therapy. The seven wounds that had not received prior antimicrobial treatment were managed empirically with a first-generation cephalosporin (cephalexin) as broad-spectrum coverage, and no modification of therapy was required following culture results due to favourable clinical progression. Among the eight cases that had received antibiotics prior to presentation, wound healing was ultimately achieved using EMA category C antimicrobials without therapeutic modification in most instances; only Cases 1, 3, and 8 required escalations to category B agents. Importantly, category A antimicrobials were not required in any patient. These findings indicate that, even in the presence of multidrug-resistant isolates and variable susceptibility profiles, effective infection control can be achieved through a combination of appropriate systemic therapy and adequate medical wound management while adhering to antimicrobial stewardship principles.

This prospective randomised split-wound clinical study evaluated the efficacy of FB as an adjunct to a standardised wound management protocol in dogs and cats with open wounds of different aetiologies. The findings did not support the initial hypothesis, as all outcome measures, including quantitative, qualitative, and semiquantitative histopathological/immunohistochemical endpoints did not demonstrate statistically significant differences between the treated and control portions over the four-week follow-up period. Overall, healing progression evolved similarly in both portions, regardless of how outcomes were assessed.

Planimetric analysis revealed a heterogeneous response pattern, with seven wounds favouring the treated portion, two showing comparable outcomes between portions, and six favouring the control portion. Despite this numerical distribution, paired analyses demonstrated no statistically significant difference in percentage wound area reduction between treated and control portions. Histopathological and immunohistochemical findings were consistent with the clinical outcomes, as no significant differences were detected between treated and control portions in inflammatory response, collagen deposition, angiogenesis, or cellular proliferation. These findings should be interpreted cautiously, since biopsies were only available from a limited subset of surgically managed wounds (seven wounds, with one wound excluded from paired histological comparisons) and were collected at the time of closure, when earlier transient biological effects may no longer have been detectable. Consequently, these endpoints should be considered exploratory, and the histological and immunohistochemical findings should be regarded as hypothesis-generating rather than definitive evidence of treatment-related biological effects.

Although no histological or immunohistochemical variable reached statistical significance, only small numerical differences were observed between treated and control portions. Composite histological scores, collagen deposition, and angiogenesis scores were slightly higher in control portions, whereas fibroblast proliferation was marginally higher in treated portions. CD31 expression remained very low in both portions, and Ki67 scores were slightly lower in treated samples. These subtle and somewhat inconsistent tissue-level findings suggest that any biological effect, if present, was limited and not clearly detectable under the conditions tested.

Several methodological and biological factors may have limited the ability to detect subtle or localised treatment effects in the present study. Although the planned sample size was reached, the study may still have been underpowered to detect small incremental effects in a heterogeneous clinical population, particularly given informative censoring due to early closure/surgery and interobserver variability in ordinal scoring. Accordingly, the absence of statistically significant differences should be interpreted with caution, as small treatment effects may have remained undetected within this clinically diverse population. Consistent with this interpretation, effect size estimates for both the primary and secondary outcomes were negligible to small, and all confidence intervals included zero, although their width reflects the uncertainty inherent to the limited sample size and clinical heterogeneity of the study population.

These findings therefore suggest that, under the conditions tested, any potential clinical benefit of FB is likely modest or restricted to selected wound presentations. Nevertheless, the relatively small sample size in absolute terms, combined with the marked heterogeneity of wound aetiologies, anatomical locations, and patient characteristics, likely increased variability and reduced the ability to detect small effect sizes. This heterogeneity included differences in wound duration, underlying causes, previous treatments, and concurrent medical conditions, all of which may have contributed to biological variability. One dog included in the study was diagnosed with leishmaniosis. Infectious and systemic diseases may influence wound healing through alterations in immune function, inflammation, tissue perfusion, and cellular repair mechanisms. In particular, canine leishmaniosis is associated with complex immune dysregulation that may affect tissue homeostasis and repair processes. In this case, treatment for leishmaniosis was initiated concurrently with standardised wound management, which may have mitigated any potential negative impact of the disease on wound repair (43, 44). However, given that only a single affected animal was enrolled, no conclusions can be drawn regarding the influence of leishmaniosis on wound healing or response to fluorescence biomodulation.

In addition, the number of evaluable cases declined over time due to wound closure or surgical intervention, further limiting longitudinal comparisons. Finally, interobserver variability—particularly in the assessment of necrosis and inflammation—may have contributed additional measurement noise, potentially obscuring minor differences between wound portions despite the use of blinded observers and a structured scoring system. Furthermore, the macroscopic scoring system was specifically developed for this study based on the TIME framework and had not undergone prior formal validation; therefore, the observed agreement analyses should be interpreted as exploratory measures of observer consistency rather than validation of the scoring instrument itself.

Similarly, only modest interobserver agreement was observed for the overall histological and immunohistochemical scores, highlighting the inherent subjectivity of semiquantitative scoring systems and the difficulty of detecting subtle biological changes in small exploratory clinical datasets.

An additional consideration is the potential for partial crossover effects in smaller wounds. In several cases, the total wound surface area was limited (<7 cm^2^), and the treated and control portions were separated only by a narrow interface. Under these conditions, fluorescence-generated photobiological effects may have extended beyond the intended treated region, attenuating detectable differences between portions. This phenomenon may have reduced the biological contrast between treatment groups and consequently limited the ability of the study to detect small treatment-related effects. Future studies should address this limitation by including larger wounds or increasing the physical separation between treated and untreated area.

Differences in biological context and dose requirements may also have contributed to the observed findings. While currently recommended FB dosing regimens have been associated with clinical improvement in dermatological conditions such as interdigital furunculosis, superficial or deep pyoderma, pyotraumatic dermatitis, and perianal fistula—where inflammation is generally more superficial and tissue architecture remains relatively preserved (22, 24–27)—open wounds involve deeper tissue disruption, greater devitalization, and more complex remodelling processes that may require higher energy doses, longer exposure times, or increased treatment frequency to elicit a measurable biological response (39, 45, 46). Despite the widespread use of PBMT in animals, standardised treatment protocols remain lacking, and its clinical efficacy continues to be debated (34). Early *in vitro* studies demonstrated enhanced healing of cutaneous wounds and burns in mice (35), whereas subsequent investigations using similar parameters in deeper tissues reported negative outcomes, raising concerns about its effectiveness (36). These discrepancies are now largely attributed to inadequate devices or suboptimal therapeutic parameters, underscoring the importance of selecting protocols based on the photonic dose reaching the target tissue (37, 46). Key determinants of laser dose and clinical response include wavelength, which governs penetration depth; spot size, defining the illuminated area; power, representing the energy delivered per second; and application time (47). Together, these parameters determine irradiance and fluence, driving the biological response (47), which is dose-dependent and follows the biphasic Arndt–Schulz law, whereby insufficient stimulation produces no effect while excessive doses may inhibit tissue repair (15).

Considered collectively, these elements suggest that the limited separation between portions, the depth and severity of tissue injury, and the absence of standardised dose optimization may have reduced the likelihood of detecting marked differences between treated and control regions within the duration of this study.

Because final percentage area reduction at a single endpoint may be insensitive to differences in healing kinetics when both portions improve under an effective standard-of-care protocol, exploratory analyses were conducted to evaluate the temporal pattern of wound area reduction. A time-integrated metric based on the AUC of normalised wound area suggested a directional trend favouring the treated portion in 8 of 15 cases. Although this difference was not statistically significant, it indicates that, in slightly more than half of the cohort, the treated portion spent less time with a larger open surface area during follow-up. When stratified by wound duration at inclusion, this directional pattern was more consistent in wounds present for ≤7 days of presentation. Because these analyses were performed *post hoc* and were not predefined study endpoints, they should be interpreted cautiously and considered exploratory observations rather than evidence of a definitive biological effect. While post hoc and hypothesis-generating, this finding is biologically plausible, as photobiomodulation effects may be more pronounced during the early inflammatory and proliferative phases of wound repair, whereas established wounds may be dominated by factors such as devitalized tissue burden, bacterial contamination, impaired perfusion, and mechanical constraints. This interpretation is supported by extensive evidence demonstrating beneficial effects of PBMT in both human and veterinary dermatologic conditions, including otitis externa, canine interdigital and deep pyoderma, and perianal fistula (22–27). Within the context of wound healing, positive outcomes have been reported in chronic, post-surgical, and diabetic wounds (28–30). In dogs specifically, improved collagen deposition, reduced inflammation, and enhanced healing have been described in incisional wounds treated with FB (32), although other studies involving surgically created and open wounds have failed to demonstrate significant benefits following laser therapy (31, 33). Similarly, conflicting findings have been reported in veterinary dentistry, with one study showing no improvement in healing time or scarring after palatal surgery and another documenting clinically meaningful improvements in healing and pain scores (48, 49). Collectively, this heterogeneous body of evidence suggests that PBMT effects may be context-dependent, potentially explaining why a temporal advantage was observed primarily in more recent wounds within the present study.

A clinically meaningful milestone—complete wound closure within the observation period—was also explored. Closure occurred exclusively in the treated portion in three cases and exclusively in the control portion in one case, with additional wounds achieving closure in both portions. Although these differences were not statistically significant, the imbalance among discordant pairs may suggest that any potential benefit of FB, when present, could manifest as earlier attainment of clinically relevant endpoints in selected wounds rather than as a marked divergence in final percentage area reduction at a fixed time point.

In summary, while outcome measures of this split-wound study did not demonstrate a consistent measurable advantage of FB therapy over standardised wound management alone, exploratory time-integrated analyses indicated a directional trend toward earlier wound area reduction in some cases, particularly when treatment was initiated early. Accordingly, the findings suggest that any potential benefit, if present, may be modest and limited to selected wound presentations, and should be evaluated in prospectively stratified studies with endpoints focused on healing speed and readiness for closure.

## Data Availability

The original contributions presented in the study are included in the article/Supplementary material, further inquiries can be directed to the corresponding author.
