# Supplementary material for: Effect of fluorescence biomodulation as an adjunct to standard wound management in open wounds in dogs and cats: a prospective, randomised, within-wound controlled clinical trial
Source: Front Vet Sci. 2026 Jul 22;13:1870940. doi: 10.3389/fvets.2026.1870940 (PMC13437437; doi:10.3389/fvets.2026.1870940)
Supplement: Supplementary file 1 [file Data_Sheet_1.PDF]

Supplementary Material

1 Supplementary Tables

1.1 **Supplementary Table 1 (S1):** Bacterial isolates and antimicrobial susceptibility according to EMA categories. EMA antimicrobial categories: A ("Avoid"), B ("Restrict"), C ("Caution"), D ("Prudence").

Case	Bacterial isolates	EMA categories with susceptibility to all bacterial isolates	Susceptible antimicrobials to all bacterial isolates
1	*Enterococcus faecalis*, *Pseudomonas aeruginosa*,	B	Marbofloxacin,
2	*Pseudomonas aeruginosa*, *Escherichia coli*	A, B, C	Imipenem, enrofloxacin, marbofloxacin, ciprofloxacin, gentamicin, amikacin, ciprofloxacina
3	*Enterobacter hormaechei*	C	Amikacin
4	*Acinetobacter calcoaceticus*, *Pseudomonas monteilii*	A, B, C, D	Ceftazidime, imipenem, marbofloxacin, ciprofloxacin, gentamicin, amikacin, doxycycline
5	*Enterococcus faecalis*, *Staphylococcus aureus*	B, C	Fluoroquinolones, erythromycin
6	*Enterobacter hormaechei*	A, C	Ceftazidime, imipenem, gentamicin, amikacin
7	*Acinetobacter spp.*, *Proteus mirabilis*	A, B, C, D	Ceftazidime, imipenem, fluoroquinolones, TMP-SMX, gentamicin, amikacin
8	*Pseudomonas aeruginosa*	A, B, C	Ceftazidime, imipenem, enrofloxacin, marbofloxacin, ciprofloxacin, gentamicin, amikacin
9	*Enterobacter hormaechei*, *Escherichia coli*, *Pseudomonas monteilii*	C, D	Gentamicin, amikacin, doxicicline
10	*Enterobacter hormaechei*, *Escherichia coli*, *Pseudomonas monteilii*	C, D	Gentamicin, amikacin, doxicicline
11	*Klebsiella oxytoca*, *Escherichia hermannii*, *Pseudomonas mendocina*	A, B, C, D	Ceftazidime, Ciprofloxacina,imipenem, fluoroquinolones, gentamicin, amikacin, TMP-SMX, doxicicline
12	*Pseudomonas aeruginosa*, *Pseudomonas putida*	B, C	Ceftazidime, fluoroquinolones, gentamicin, amikacin
13	*Streptococcus canis*	B, C, D	Penicillins, cephalosporins, fluoroquinolones, TMP-SMX, clindamycin, erythromycin, tetracyclines
14	*Staphylococcus felis*, *Staphylococcus pseudintermedius*, *Streptococcus canis*	B, C, D	Amoxicillin–clavulanic acid, cefalotin, cefpodoxime, cefovecin, TMP-SMX, clindamycin, erythromycin, chloramphenicol, florfenicol, tetracyclines, doxicicline
15	*Staphylococcus aureus*, *Candida spp.*	B, C, D	Fluoroquinolones, TMP-SMX, amikacin, clindamycin, erythromycin

1.2 Supplementary Table 2 (S2): Results of the quantitative assessment of wound surface area (cm²) using digital planimetry software. C = Control portion; T = Treated portion; S = Week.

CASE	Cs1	Cs2	Cs3	Cs4	% REDUCTION C	Ts1	Ts2	Ts3	Ts4	% REDUCTION T
**1**	34.06	18.38	10.52	3.28	90.4	22.7	21.91	18.23	13.94	38.3
**2**	11.66	7.42	4.4	2.81	75.9	19.95	10.52	3.42	1.15	94
**3**	54.25	49.66	33.76		37.8	23.62	26.58	18.07		23.5
**4**	4.7	5.8	3.96	1.7	63.8	4.7	6.69	1.82	0.45	89.4
**5**	15.4	6.4	4.4	1.69	89.1	14.8	7.9	7.9	5.4	63.5
**6**	13.62	9.23	3.8		72.1	14.4	3.32	0		100
**7**	4.3	1.48	0.78		81.9	2	0.28	0		100
**8**	3.1	2.8	1.25	0.42	86.5	3.1	3.6	1.94	1.4	54.8
**9**	2.45	2.23	0		9.0	2.79	0.33	0		88.2
**10**	1.2	0.51			57.5	1.5	0			100
**11**	1.03	0.65	0		100	1.12	0.37	0		100
**12**	2.97	1.81	1.41	1.43	51.9	3.43	4.78	2.8	2.7	21.3
**13**	3.25	6.02	3.07		5.5	6.64	4.02	3.86		41.9
**14**	5.15	1.48	0.58	0	100	6.15	2.95	1.24	0	100
**15**	3.58	1.45	0.64	0	100	7.32	3.25	1.36	1.1	84.9

1.3 Supplementary Table 3 (S3): Qualitative wound scores by week – Control portions. Values are presented as mean ± SD of the averaged scores assigned by two blinded observers. Lower scores indicate better wound healing. Missing values due to complete wound closure were considered non-evaluable and excluded from weekly summaries.

Week	n	Necrotic tissue	Inflammation/Infection	Granulation tissue	Epithelialization
1	15	0.17 ± 0.31	1.53 ± 0.40	2.47 ± 0.58	2.77 ± 0.26
2	15	0.03 ± 0.13	1.37 ± 0.35	1.60 ± 0.63	1.90 ± 0.66
3	12	0.00 ± 0.00	0.88 ± 0.38	1.33 ± 0.49	1.42 ± 0.60
4	8	0.00 ± 0.00	0.75 ± 0.65	1.00 ± 0.53	1.00 ± 0.60

1.4 **Supplementary Table 4 (S4):** Qualitative wound scores by week – Treated portions. Values are presented as mean ± SD of the averaged scores assigned by two blinded observers. Lower scores indicate better wound healing. Missing values due to complete wound closure were considered non-evaluable and excluded from weekly summaries.

Week	n	Necrotic tissue	Inflammation/Infection	Granulation tissue	Epithelialization
1	15	0.27 ± 0.46	1.50 ± 0.38	2.40 ± 0.60	2.77 ± 0.37
2	14	0.07 ± 0.18	1.46 ± 0.31	1.68 ± 0.75	2.14 ± 0.66
3	10	0.00 ± 0.00	1.00 ± 0.47	1.45 ± 0.60	1.75 ± 1.01

4	7	0.00 ± 0.00	1.07 ± 0.53	1.50 ± 0.76	1.36 ± 0.63

1.5 **Supplementary Table 5 (S5):** Supplementary Table 5 (S5): Summary of semiquantitative tissue-based assessments in surgically managed wounds. C: control portion; T: treated portion; NA: non-evaluable sample. *Case 10 was excluded from paired histology and trichrome comparisons due to a non-evaluable treated sample. Histology Total represents the mean composite histological score assigned independently by two blinded observers (inflammation + fibroblast proliferation + angiogenesis + collagen deposition). IHC Total represents the mean composite immunohistochemical score (CD31 + Ki67). Statistical comparisons were performed using the Wilcoxon signed-rank test.

Case	Histology Total C	Histology Total T	Trichrome C	Trichrome T	IHC Total C	IHC Total T
**2**	7.0	6.5	1.5	1.5	0.5	0.0
**3**	4.0	4.5	2.0	1.5	0.5	1.0
**5**	7.5	6.5	2.5	1.0	0.5	1.5
**6**	5.5	7.0	1.5	2.0	1.0	0.5
**9**	5.0	5.5	0.5	2.0	0.0	0.0
**10**	8.5	NA	3.0	NA	0.0	0.0
**13**	7.5	7.0	1.5	1.5	1.5	0.0

Variable	Control Mean	Treated Mean	p-value
**Histology Total***	6.43	6.17	0.914
**Trichrome***	1.79	1.58	1.000
**IHC Total**	0.57	0.46	0.665
